# Stability of immobilized L-arginine deiminase from *Penicillium chrysogenum* and evaluation of its anticancer activity

**DOI:** 10.1038/s41598-024-77795-8

**Published:** 2024-11-08

**Authors:** Hamed M. El-Shora, Gharieb S. El-Sayyad, Nessma A. El-Zawawy, Mohamed A. Abd El-Rheem, Metwally A. Metwally, Sally A. Metwally

**Affiliations:** 1 Department of Botany, Faculty of Science, Mansoura University, Mansoura, Egypt; 2 Medical Laboratory Technology Department, Faculty of Applied Health Sciences Technology, Badr University in Cairo (BUC), Cairo, Egypt; 3 Department of Microbiology and Immunology, Faculty of Pharmacy, Galala University, Galala City, Suez, Egypt; 4 Drug Microbiology Lab, Drug Radiation Research Department, National Center for Radiation Research and Technology (NCRRT), Egyptian Atomic Energy Authority (EAEA), Cairo, Egypt; 5 Department of Botany and Microbiology, Faculty of Science, Tanta University, Tanta, 31527 Egypt

**Keywords:** L-arginine deiminase, Immobilization, Chitosan, Stability, Pepsin, Anticancer activity, Biochemistry, Enzyme mechanisms, Enzymes, Cancer

## Abstract

The aim of the present work was to immobilize L-arginine deiminase on suitable supports such as chitosan, alginate, and silica gel to study its stability. Additionally, the study aims to investigate the anticancer effects of the free purified enzyme on hepatocellular carcinoma (Hep-G2) and breast cancer (MCF-7) cell lines. L-arginine deiminase (ADI: EC 3.5.3.6) was immobilized on chitosan, Ca-alginate, and silica gel, with immobilization efficiencies of 89.0%, 72.8%, and 66.5%, respectively. The optimal immobilization time for the highest efficiency was 4 h. Increasing the concentration of glutaraldehyde improved the immobilization efficiency of ADI on chitosan. The chitosan-immobilized ADI retained about 45% of its activity after 8 cycles. The optimal pH values were 6 for the free purified ADI and 7 for the chitosan-immobilized ADI. The optimal temperature increased from 40 °C for the free enzyme to 45 °C after immobilization. The activation energies for the free and chitosan-immobilized enzymes were 71.335 kJ/mol and 64.011 kJ/mol, respectively. The K_m_ values for the free and chitosan-immobilized ADI were 0.76 mM and 0.77 mM, respectively, while the V_max_ values were 80.0 U/mg protein for the free ADI and 71.4 U/mg protein for the chitosan-immobilized ADI. After 30 days of storage at 4 °C, the residual activities were 40% for the free purified ADI and 84% for the chitosan-immobilized ADI. At 25 °C, the residual activities were 10% for the free ADI and 75% for the chitosan-immobilized ADI. The chitosan-immobilized ADI exhibited significantly higher stability against proteases such as pepsin and trypsin compared to the free enzyme. The purified ADI also demonstrated enhanced potential anticancer effects and significant cytotoxicity against the Hep-G2 and MCF-7 tumor cell lines compared to doxorubicin. These findings suggest that purified ADI has potential as an anticancer agent, though further in-depth studies are required.

## Introduction

A powerful proteinogenic amino acid, L-arginine is involved in a number of biosynthesis and energy-generating processes, including the tricarboxylic acid (TCA) cycle and the urea (ornithine) cycle. Two enzymes, argininosuccinate synthetase (ASS) and argininosuccinate lyase (ASL), convert citrulline into L-arginine, a nonessential amino acid in humans. Citrulline is changed by ASS into argininosuccinate, which ASL then transforms into arginine^1^.

With a high affinity for L-arginine, L-arginine deiminase (ADI) (E.C.3.5.3.6) catalyzes the conversion of L-arginine’s guanidine group to citrulline and ammonium ions^2^. Since Horn’s 1933 study of *Bacillus pyocyaneus*, the enzyme has been found and described in a variety of microorganisms, including bacteria^3–7^ and fungi^8^.

An efficient method for enzyme-catalyzed reactions in industry is the immobilization of enzymes. Because the enzyme molecules’ conformational mobility is limited after immobilization, the main goal of enzyme immobilization is to strengthen the enzyme’s stability against a variety of deactivation pressures. Therefore, compared to the free ADI, the immobilized enzyme could function under harsh environmental conditions with less activity loss^9^. Increased resilience to temperature and pH variations is a benefit of the immobilized enzyme. Under such circumstances, enzymes stay in place during the reaction, facilitating easy product separation, ongoing functionality, and reusability^10^.

The second most common cause of mortality worldwide is cancer. Different cancers are treated with a variety of methods, such as radiation therapy, chemotherapy, and other drugs. However, because of their poor specificity, these therapies frequently have their own unique set of adverse effects. New treatments that do not impede the regular operation of healthy cells are therefore desperately needed. Depriving malignant cells of nutrients or non-essential/semi-essential amino acids, like those to which they are auxotrophic, is one promising anticancer treatment^11^.

Using arginine-depriving enzymes (ADE) like the arginase, arginine decarboxylase (ADC), and arginine deiminase (ADI) could be a useful cancer treatment approach because L-arginine is a semi-essential vital amino acid that is essential for a number of metabolic processes, signaling pathways, and the growth of cancer cells^12^. L-Arginine is an effective nitrogen source used by cancer cells for nucleic acid and protein synthesis^13^. Consequently, one way to treat certain tumours is by depleting L-arginine. Arginine-depleting enzymes such as arginine deiminase (ADI) may be more effective against cells lacking argininosuccinate synthetase (ASS). It has been discovered that human hepatocellular and melanoma cell lines are auxotrophic for arginine because they do not express ASS. According to in vitro research, arginine depletion may be useful in the treatment of certain tumors as well as maybe others^14^.

Hepatoma, aggressive fibrosarcoma, malignant melanoma, squamous cell carcinoma, lung carcinoma, and nasopharyngeal carcinoma are among the human diseases for which the conversion of L-arginine to ammonia and L-citrulline produces an anti-tumor effect both in vitro and in vivo^15^. Phase II clinical studies for hepatocellular carcinoma, malignant mesothelioma, and metastatic melanoma have shown promise using ADI^16,17^.

Globally, the incidence of breast cancer is rising, and the death rate is high^18^. Breast cancer cell lines respond differently to certain treatment drugs, indicating that breast cancer is an extensive and diverse illness^19^. Approximately 90% of instances of liver cancer are hepatocellular carcinoma (HCC), making it the most prevalent kind. Although hepatitis B and hepatitis C virus infections are the main risk factors for HCC, non-alcoholic steatohepatitis associated with metabolic syndrome or diabetes mellitus is a risk factor that is becoming more and more common^20^.

The current work’s objective was to explore the stability of ADI by immobilizing it on appropriate supports such as silica gel, chitosan, and Ca-alginate. The present study also intends to examine the beneficial impacts of the free pure enzyme on cell lines of breast cancer (MCF-7) and hepatocellular carcinoma (Hep-G2).

## Materials and methods

### Isolation and purification of fungal strain

Accession No. MN219732 for *Penicillium chrysogenum* Thom AUMC 14,100 gb was acquired through the Assiut University Mubasher Mycological Center (AUMMC), located in Assiut, Egypt. The growth of the fungus, isolation and purification of ADI were carried out as mentioned in our previous published article by El-Shora et al.^8^. The obtained specific activity of ADI in the previous article was 50 Umg^− 1^ protein with a 62.5-fold increase in purification.

### Immobilization of purified ADI on alginate bead

Foster et al. (2003) provided the model for ADI immobilization, which was first used^21^. 50 milliliters of 5% w/v sodium alginate were mixed with the purified ADI. After being moved to a separating funnel, the solution was suspended over 200 milliliters of 3% CaCl_2_ (w/v) in a beaker. A 200 µL Eppendorf tip was used to drop the alginate solution into the CaCl_2_ solution at a rate of 25 drops per minute. The produced bead was left 3 h under stirring gently to harden. The bead was filtered and washed by the same buffer followed by assessing the activity of the immobilized ADI.

### Immobilization of purified ADI on silica gel

One gram of silica gel was combined with 25 units of ADI which was purified and stored overnight at 4 °C. The bead was subsequently rinsed twice with 5.0 ml of phosphate buffer (10 mM, pH 7.0) to remove the unbounded ADI followed by assessing the ADI activity in the washing solution^22^.

### Immobilization of purified ADI on chitosan bead

One milliliter of glacial acetic acid was added to five grams of chitosan powder that had been suspended in one hundred milliliters of distilled water and swirled for twenty minutes. The mixture was then left to stir for five hours at room temperature. To counteract the acetic acid in the chitosan bead, drops of NaOH (2% w/v) were added after the solution had been filtered and dried. After being cleaned twice with distilled water, the bead was once again completely dried. To do the cross-linking, dried chitosan was added to 8% v/v glutaraldehyde solution in 100 mM cold potassium phosphate buffer (pH 7.0) for three hours. To get rid of extra glutaraldehyde, the resultant beads were washed twice with 100 mM cold potassium phosphate buffer (pH 7.0). In 150 mM potassium phosphate buffer (pH 7.0), chitosan beads and ADI solution (5 mg mL^− 1^) were mixed for 4 h at 4 °C while being gently stirred. After the beads were dried at 4 °C, the activity of the immobilized ADI was measured by deducting the activity that was transferred to the chitosan bead from the activity that was noted in the solution above following immobilization.

### Assay of the immobilized ADI

Choi et al.^23^ state that the test mixture included 0.1 gram of immobilized ADI and 0.5 ml L-arginine (100 mM) in 100 mM potassium phosphate buffer (pH 7.0). After 30 min at 45 °C, 10% (w/v) TCA was added to halt the reaction, and the mixture was centrifuged for 10 min at 6000 g. After mixing 0.5 ml of the supernatant with 0.2 ml of Nessler’s reagent and waiting 10 min, the color was measured at 500 nm. Under typical assay conditions, one unit of ADI was defined as the amount of ADI required to release one µmol of NH_4_ per minute^24^.

### Determinations of immobilization efficiency for ADI

The following formula was used to calculate the immobilization effectiveness of ADI:

$${\text{Immobilization efficiency }}\left( \% \right)=\left( {{\text{Activity of immobilized enzyme}}/{\text{Activity of free enzyme used}}} \right) \times {\text{1}}00$$

### Effect of the time and glutaraldehyde concentration on ADI immobilization efficiency using chitosan

The influence of immobilization time (1, 2, 3, 4, 5 and 6 h) on immobilization efficiency of ADI was investigated. At different concentrations (4, 8, 12, 16, 20 and 24% v/v), the impact of glutaraldehyde on immobilized ADI performance was examined in the reaction medium and the immobilization efficiency of ADI was determined.

### Reuse of chitosan-immobilized ADI

After measuring the immobilized ADI’s activity for eight cycles, the residual activity was calculated.

### Optimal pH and temperature of the free and chitosan-immobilized ADI

The impact of varying temperatures (20, 25, 30, 35, 40, 45, and 50 °C) and pH levels (3, 4, 5, 6, 7, 8, 9, and 10) on the free and immobilized ADI was examined. The other factors influencing ADI activity were continuously monitored.

### Determination of activation energy (Ea) of the free and chitosan-immobilized ADI

The following formula was used to determine the energy needed for activation (Ea) of the free and immobilized ADI^25^:

$${\text{Slope}}=\left( { - {{\text{E}}_{\text{a}}}/{\text{R}}} \right),{\text{where R is gas constant}}$$

### Determination of ADI kinetics

K_m_ and V_max_ of the free and immobilized ADI Lineweaver–Burk plot was calculated from using (S^− 1^) against (V^− 1^).

### Storage stability of free and chitosan-immobilized ADI at 4 °C and 25 °C

For 30 days, the free and immobilized ADI were stored at 4 °C and 25 °C. The relationship between the storage length and the residual activity (%) was plotted after the enzyme performance was assessed at 5, 10, 15, 20, 25, and 30 day intervals.

### Effect of trypsin and pepsin on free and chitosan-immobilized ADI

The effect of different stomach enzymes on ADI activity including trypsin and pepsin were tested using 10 units concentration for each enzyme at various time intervals (10, 20, 30, 40, 50 and 60 min), followed by assessing the enzyme activity.

### Antitumor activity of the purified ADI

In this investigation, cell lines from hepatocellular carcinoma (Hep-G2), breast cancer, and normal cells were employed. With or without the test substances, these cells were cultivated at 37 °C in a CO_2_ incubator in RPMI-1640 media supplemented with 10% fetal bovine serum. The MTT test was used to evaluate cytotoxicity using the Singh & Banik technique^26^. 0.1 ml of RPMI medium was used to seed cancer cells (5 × 10^4^) in every hole of 96-well plates. After 24 h, L-arginine deiminase at different doses (76.56–1250 µg mL^− 1^) was applied. Cell viability was evaluated after 48 h when 10 µL of MTT (3-(4,5-dimethylthiazol-2-yl)-2,5-diphenyl tetrazolium bromide, 5 mg mL^− 1^ stock solution; Sigma) was incorporated to each well.

After discarding the media from each well, 100 µL of DMSO was used to dissolve the formazan blue crystals that had developed in the cells. A spectrophotometer set to 570 nm was used to measure the rate of colour creation. Every experiment was carried out in a typical laboratory setting. For each tumour cell line containing the designated enzyme, a viability curve was created by charting the connection among cell survival and L-arginine deiminase concentration. In micrograms per milliliter, the effective dosage needed to 50% inhibit cell growth (IC_50_) was calculated.

### Statistical analysis

The mean ± standard deviation (SD) of three experiments is used to express the data. The statistical components across groups are shown by different letters using post hoc Duncan’s test (*p* < 0.05) and one-way ANOVA.

## Results

### Immobilization of ADI from *P. chrysogenum* on different beads

**Fig. 1:**
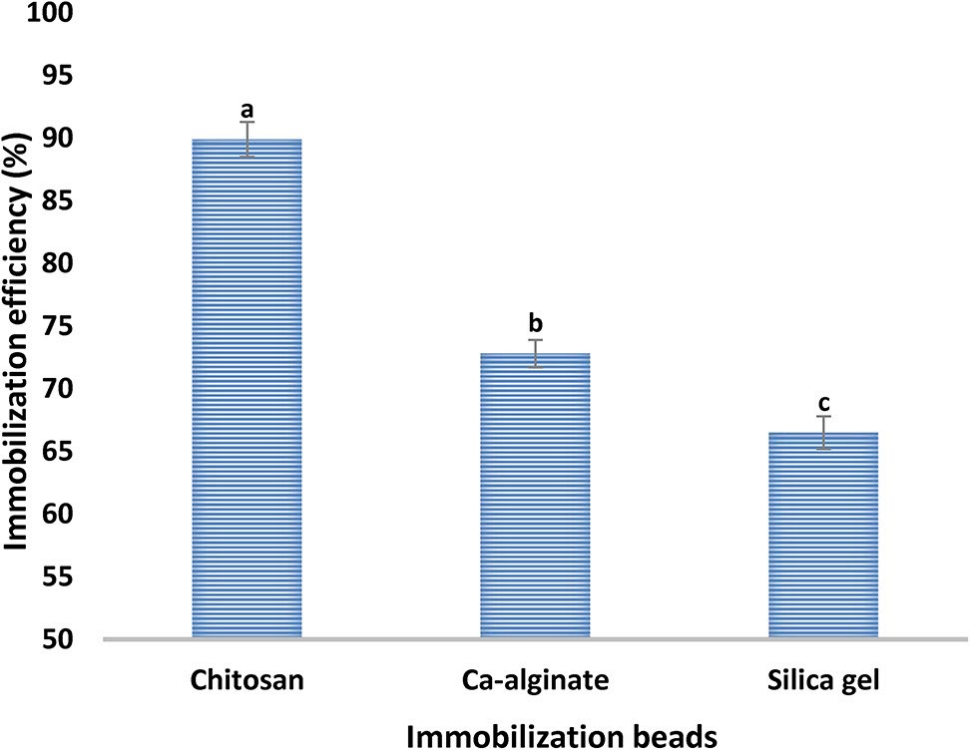
Immobilization of ADI from *P. chrysogenum* on different beads.

Arginine deiminase was immobilized on chitosan, Ca-alginate and silica gel. The results in Fig. 1 show that the immobilization efficiency values were 89.0%, 72.8% and 66.5% for the three beads, respectively. Thus, chitosan proved to be the best bead for enzyme immobilization.

### Effect of time on immobilization efficiency of ADI

**Fig. 2:**
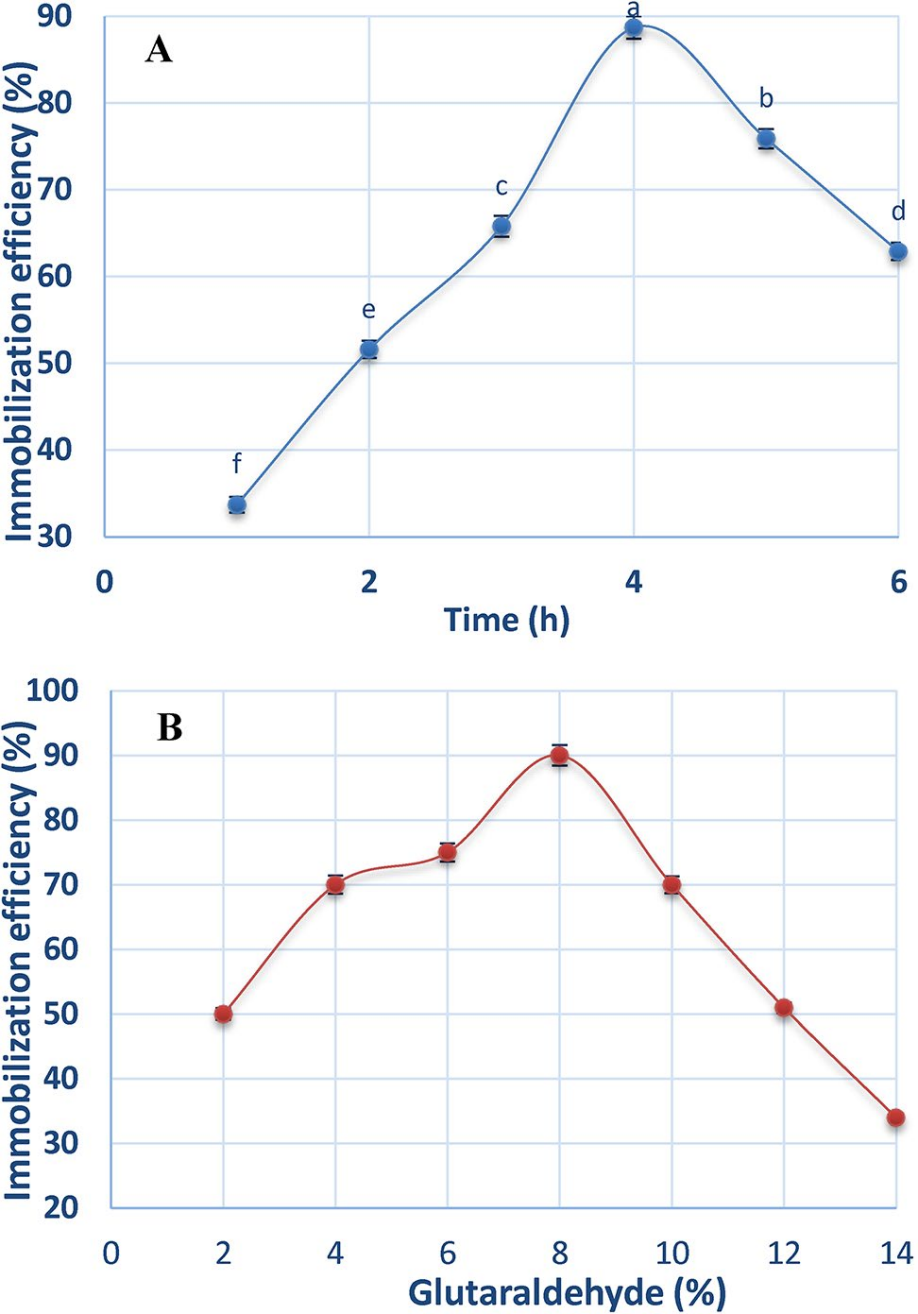
**A** Effect of immobilization time on immobilization efficiency of ADI on chitosan, **B** Effect of glutaraldehyde on immobilization efficiency of ADI on chitosan.

The effect of immobilization time on the efficiency of ADI immobilization was studied over a period of 6 h. Figure 2A illustrates that the immobilization efficiency increased gradually by increasing the time until it reached 88.7% after 4 h then declined to 75.9% and 62.9% after 5 and 6 h, respectively.

### Effect of glutaraldehyde on immobilization efficiency of ADI

The effect of glutaraldehyde on immobilization efficiency of ADI was studied at various concentrations (2, 4, 6, 8, 10, 12 and 14% v/v). The results in Fig. 2B reveal an increase in the immobilization efficiency of ADI at the lower concentrations of glutaraldehyde (2, 4, 6 and 8%) and 8% was the optimum concentration (90%). However, at the higher concentrations (10, 12, and 14%) it was observed that the immobilization efficiency declined gradually until it reached 34%.

### Reuse of chitosan-immobilized ADI

**Fig. 3:**
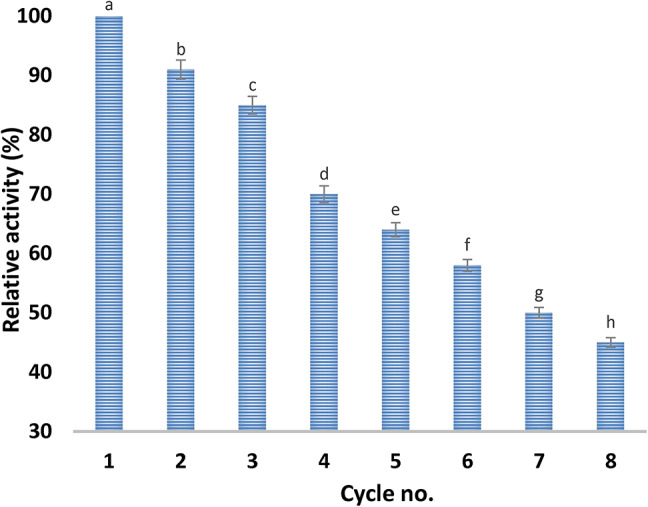
Reuse of immobilized ADI on chitosan.

By monitoring ADI activity throughout the course of eight cycles, the immobilized ADI’s reusability was examined. As the number of cycles increases, the level of activity of the immobilized ADI gradually decreases, according to the data displayed in Fig. 3. Nevertheless, the enzyme maintained 45% of its original activity following the eighth cycle.

### Effect of pH on free and chitosan-immobilized ADI

**Fig. 4:**
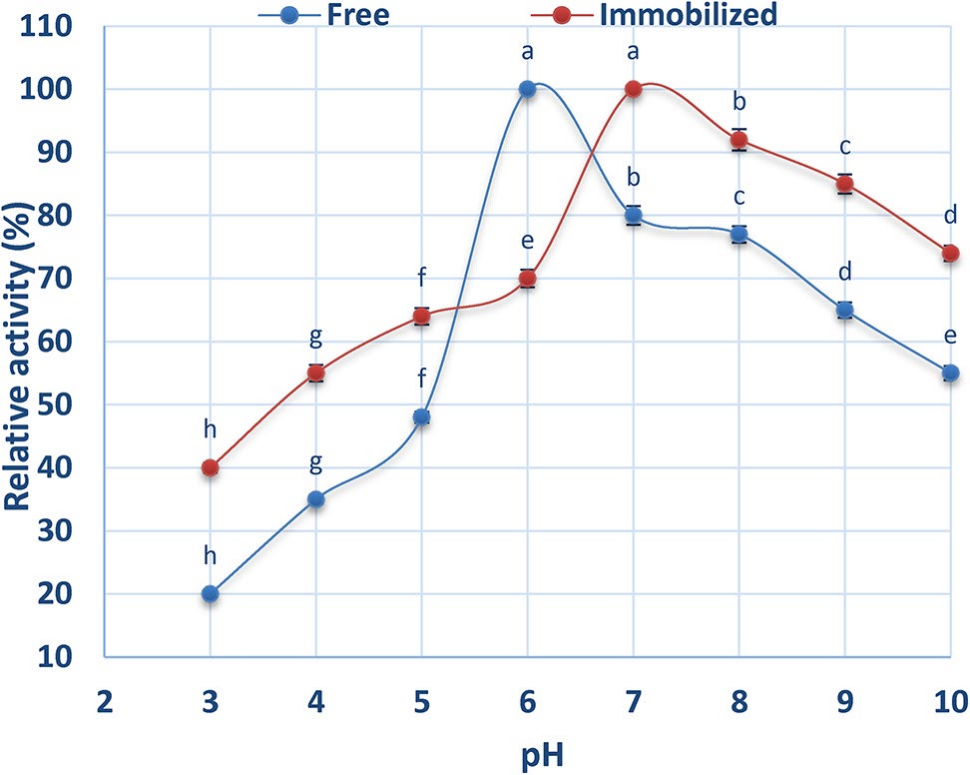
Effect of pH on free and chitosan-immobilized ADI.

The effect of pH on the activity of free and immobilized ADI was examined over a range of pH 3 to 10. According to the findings shown in Fig. 4, the optimal pH for free ADI was 6.0, whereas it shifted to pH 7.0 for immobilized ADI. It was observed that activity declined when the pH deviated from the optimal value.

### Effect of temperature on free and chitosan-immobilized ADI

**Fig. 5:**
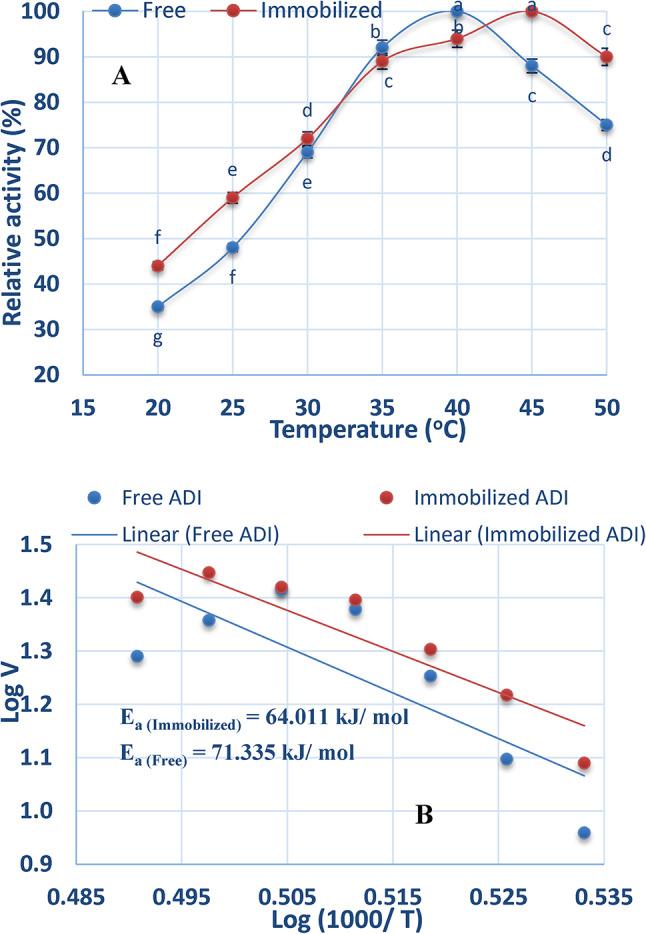
Effect of different temperatures on free and chitosan-immobilized ADI (**A**) and **Ea** of free and chitosan-immobilized ADI (**B**).

The impact of temperature on the activity of free and immobilized ADI was examined across temperature ranging from 20 to 80 °C. According to the findings shown in Fig. 5A the optimal temperature for free ADI was 40 °C, which shifted to 45 °C after immobilization. Any further rise in the temperature above the optimal one resulted in reduction of ADI activity.

Plotting the relationship between Log V against Log (1000/T) (Fig. 5B) yielded a straight line with slopes of − 8.58 and − 7.70 for the free and immobilized ADI, respectively. The calculated activation energies were 71.335 for the free ADI and 64.011 kJ/ mol for the immobilized enzyme.

### The Lineweaver–Burk plot for free and chitosan-immobilized ADI

**Fig. 6:**
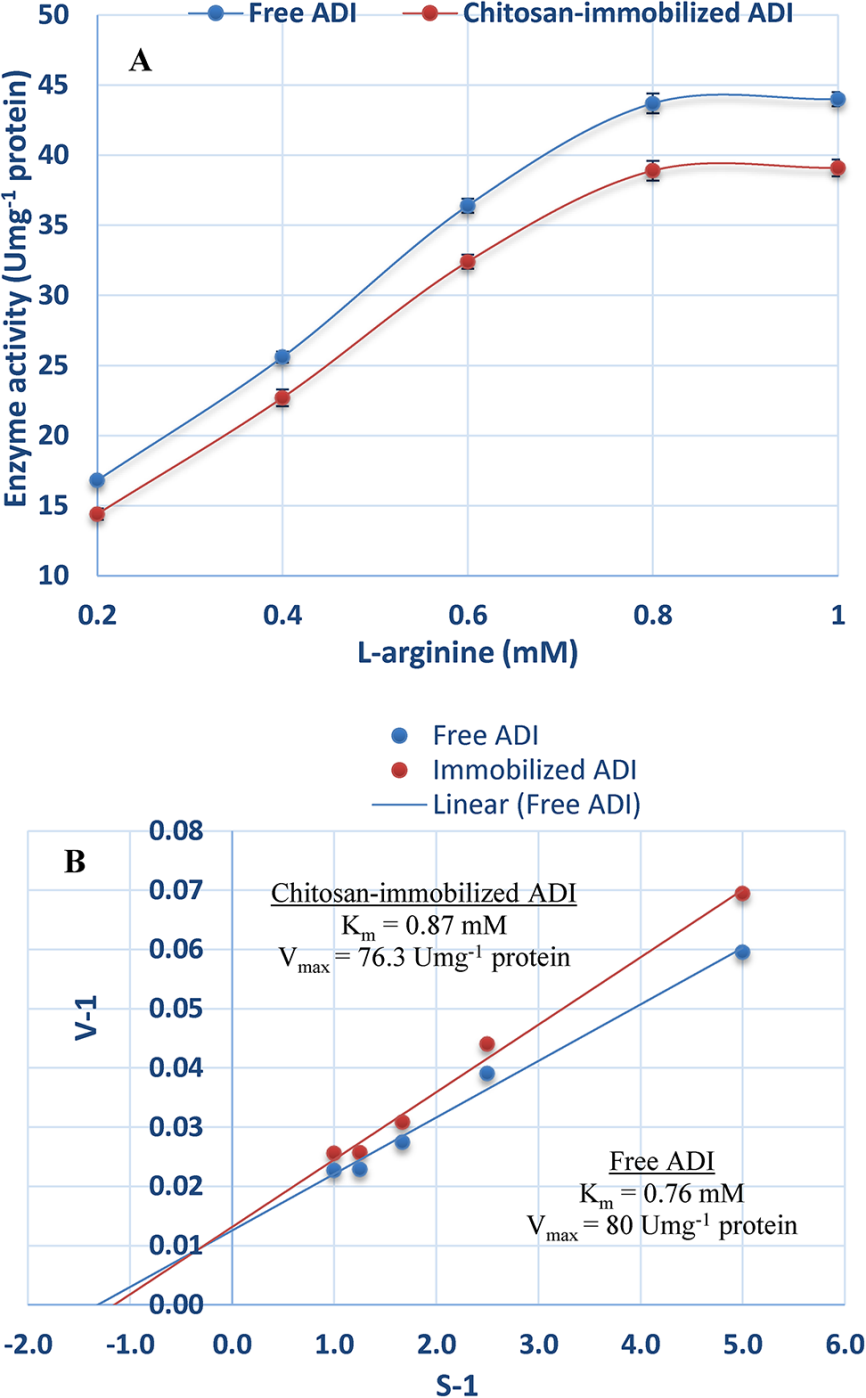
Effect of substrate concentration on free and chitosan-immobilized ADI (**A**) and the Lineweaver–Burk plot of free and chitosan-immobilized ADI (**B**).

Plotting S^− 1^ against V^− 1^ for each form of ADI (Fig. 6) produced straight line with K_m_ of 0.76 mM and V_max_ of 80.0 Umg^− 1^ protein for free enzyme. However, the immobilized ADI exhibited K_m_ of 0.87 mM and V_max_ of 76.3 Umg^− 1^ protein.

### Storage stability of free and chitosan-immobilized ADI at 4 °C and 25 °C

**Fig. 7:**
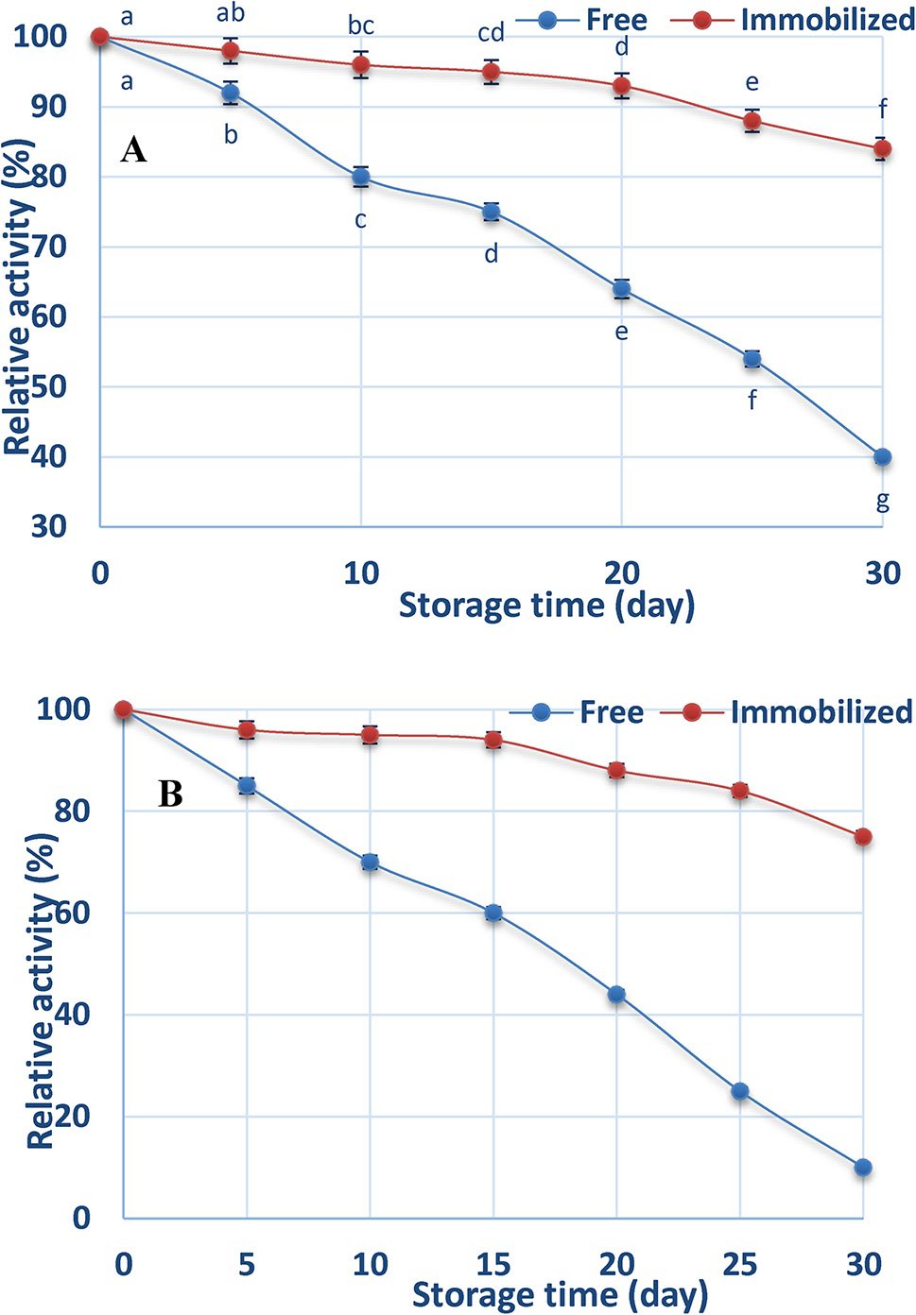
Storage stability of free and chitosan-immobilized ADI at 4 °C (**A**) and 25 °C (**B**).

Over the course of 30 days, the durability of storage of both free and immobilized ADI was assessed at 4 °C. Compared to the control value, the enzyme performance is shown. The activity level of both free and immobilized ADI gradually decreased, as indicated by the data displayed in Fig. 7A.

In contrast to the free enzyme, the immobilized enzyme continued to exhibit greater activity. The immobilized ADI maintained 84% of its original activity after 30 days, but the free ADI only maintained 40%.

At 25 °C, the activity of the two types of free and immobilized enzymes steadily decreased, as seen in Fig. 7B. However, compared to the immobilized ADI, the free ADI’s activity reduction was more noticeable. The free enzyme only kept 10% after 30 days, but the immobilized enzyme kept 75%.

### Effect of trypsin and pepsin on the activity of free and chitosan-immobilized ADI

**Fig. 8:**
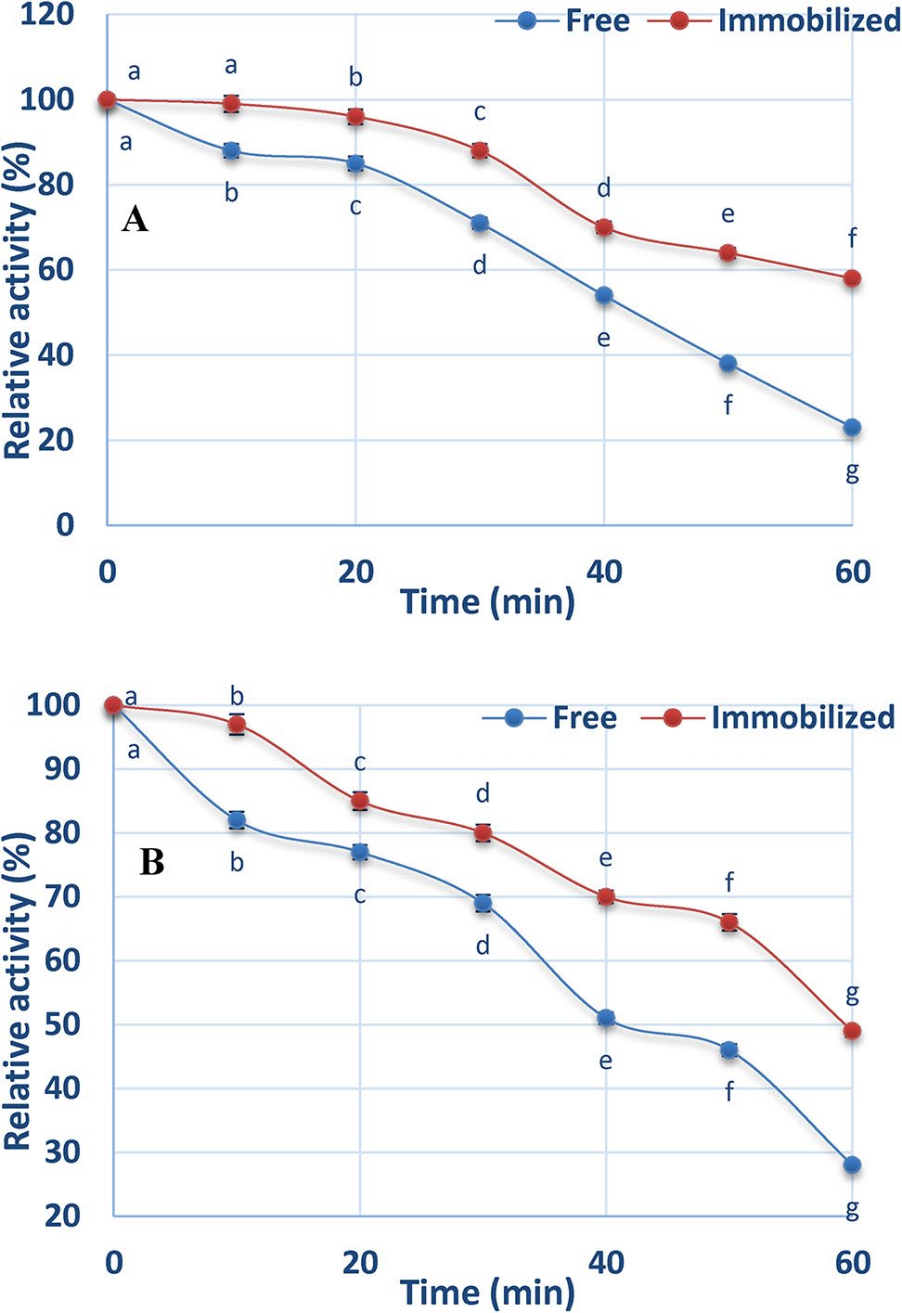
Effect of trypsin (**A**) and pepsin (**B**) on the activity of free and chitosan-immobilized ADI.

Figure 8A illustrates that the activity of both free and immobilized enzymes decreased progressively following the treatment of ADI with 10 units of trypsin, with the rate of decline being dependent on the duration of incubation.

After 60 min, the immobilized ADI retained 58% of its initial activity, whereas the free enzyme retained only 23%. Figure 8B shows that treatment with pepsin resulted in a similar pattern of activity reduction for both the free and immobilized ADI as observed with trypsin. But contrasted to the free enzyme, the immobilized enzyme showed more resistance to pepsin.

### Effect of purified ADI on cancer cell line

**Fig. 9:**
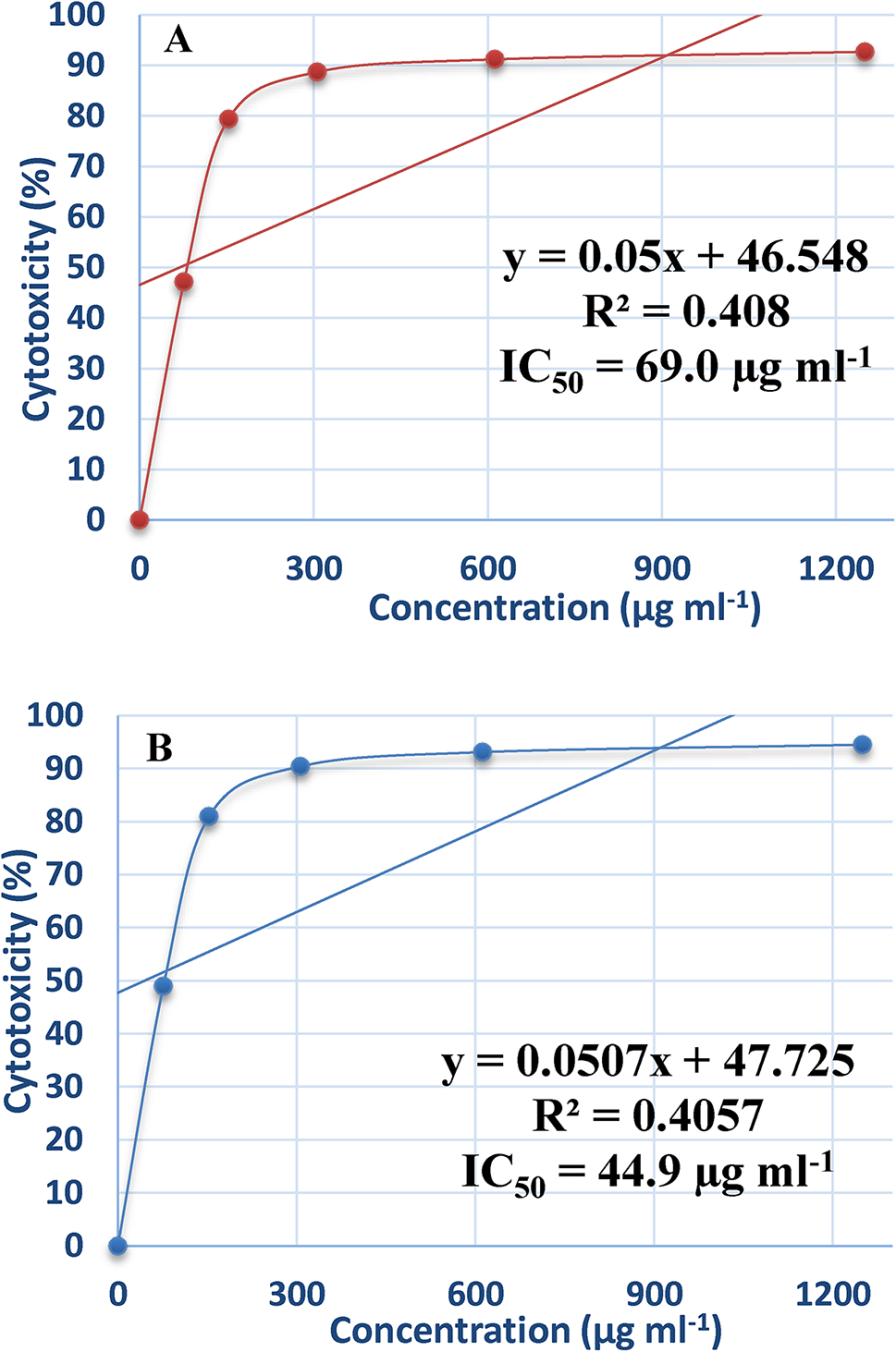
Effect of the purified ADI (**A**) and doxorubicin (**B**) on hepatocellular carcinoma (HeP-G2) cell line.

**Fig. 10:**
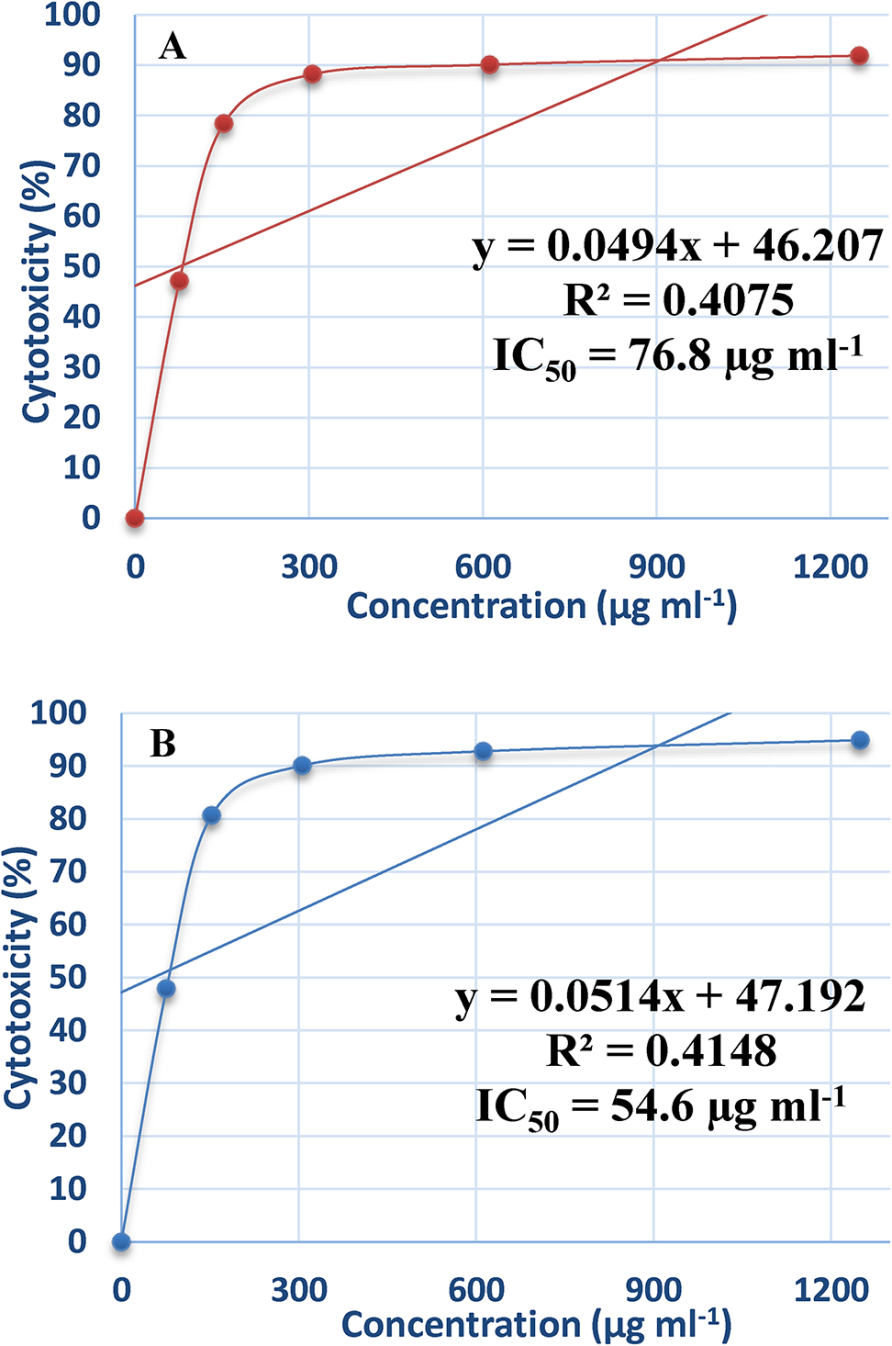
Effect of the purified ADI (**A**) and doxorubicin (**B**) on breast cancer cells (MCF-7) cell line.

**Fig. 11:**
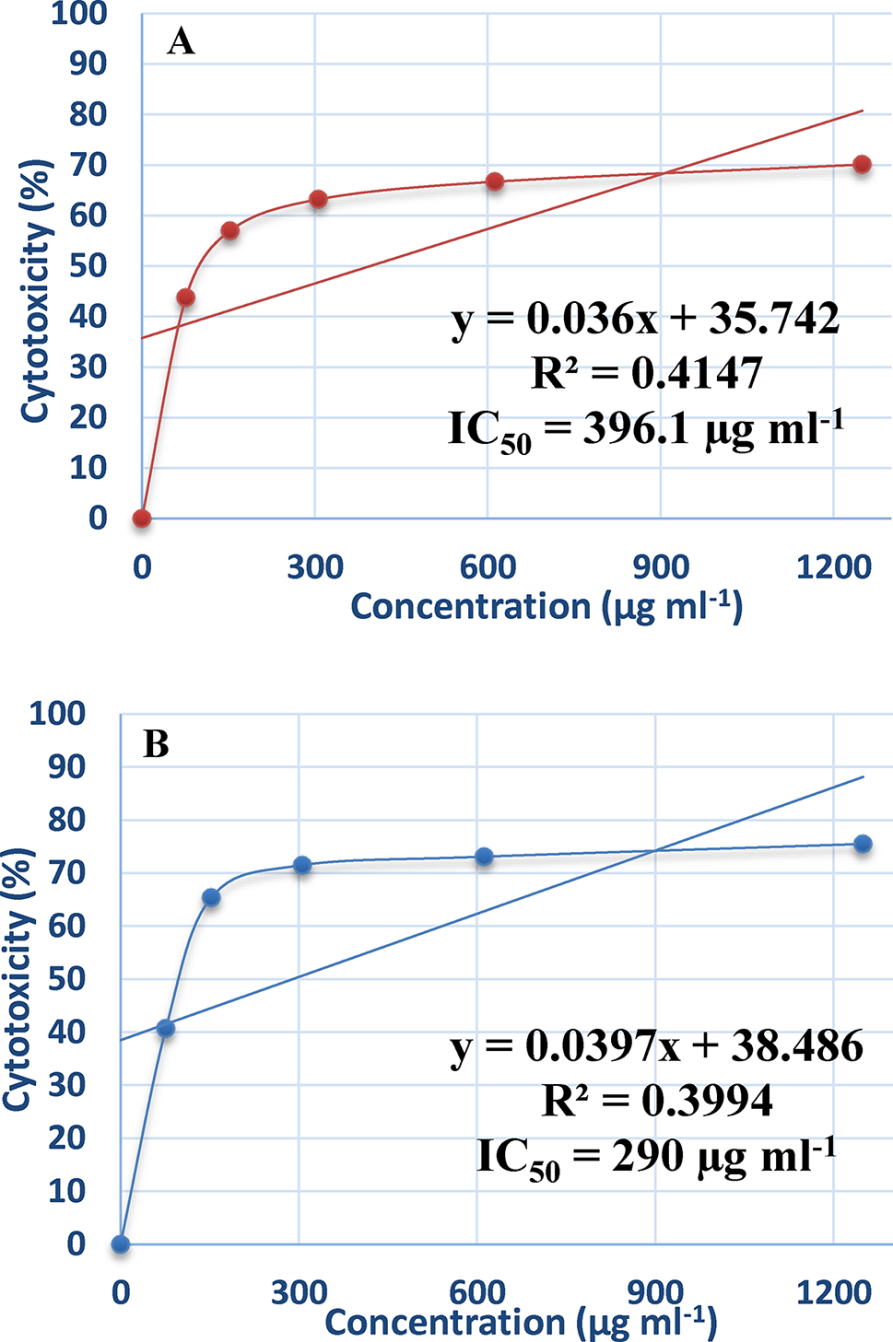
Effect of the purified ADI (**A**) and doxorubicin (**B**) on normal cell line (Wi-38).

Cancer is one of the leading causes of mortality in the world. Numerous clinically effective anti-cancer agents have been obtained from natural sources^27^. Globally, the incidence of breast cancer is rising, and significant death rates are also present^18^. Breast cancer cell lines respond differently to certain treatment drugs, demonstrating the disease’s complexity and heterogeneity^19^. In the presence of varying enzyme doses, the cytotoxic impact of L-arginine deiminase on the proliferation of normal cells (Wi-38), hepatocellular carcinoma (Hep-G2), and breast cancer cells (MCF-7) were examined. The results, shown in Figs. 9 and 10, and 11, demonstrate that incubation of Hep-G2 (Fig. 9A) and MCF-7 (Fig. 10A) cell lines with the purified L-arginine deiminase led to gradual inhibition of cell growth, with IC_50_ values of 69 µg mL^− 1^ and 76.8 µg mL^− 1^, respectively. In comparison, doxorubicin, used as a standard drug, exhibited IC_50_ values of 44.9 µg mL^− 1^ for Hep-G2 (Fig. 9B) and 45.6 µg mL^− 1^ for MCF-7 (Fig. 10B). However, the IC_50_ values for the normal cell line (Wi-38) in the presence of the purified ADI (Fig. 11A) and doxorubicin (Fig. 11B) were 369.1 µg mL^− 1^ and 290 µg mL^− 1^, respectively, which are higher than those recorded for Hep-G2 and MCF-7.

## Discussion

The purified ADI was immobilized on chitosan, Ca-alginate and silica gel. Since chitosan expressed the better immobilization efficiency, it was used for immobilization and characterization of the ADI in the present work. Chitosan has the advantages of good biocompatibility, biodegradation, non-antigenicity, non-toxicity, low cost and abundant resources. Consequently, chitosan proves to be a promising organic material for enzyme immobilization^28^. Enzyme stiffness increases with immobilization, and this is often reflected in increased stability toward denaturation when the temperature is raised^29^. It should be mentioned that stabilizing enzyme confirmation may also be significantly impacted by the support’s biocompatibility. The benefits of immobilization in terms of preservation, recycling, ongoing operation, and product purification have made it a preferred approach for large-scale enzyme applications^30^.

Addition of glutaraldehyde at various concentrations reveals continuous increase in immobilization efficiency depending on the concentration up to 8% (v/v) after which the immobilization efficiency declined at higher concentrations of glutaraldehyde. This decline is probably caused by the interaction between ADI’s amine and aldehyde groups, which encourages protein chain cross-linking. This blocks the enzyme’s active site and causes enzyme deactivation during the stabilization phase. Similar outcomes have been seen when glutaraldehyde is used to stabilize catalase^31^.

Although the enzyme particles are uniformly distributed throughout the immobilization bead, aggregation formation may cease after immobilization. In this instance, glutaraldehyde most likely creates intermolecular connections that make the enzyme preparation more stable^32^. Up to four hours, the quantity of ADI absorbed onto the support rose proportionately; after that, it decreased as the length of time spent in presence of 8% (v/v) glutaraldehyde increased.

ADI kept 45% of its activity after 8 cycles and the reusability of immobilized ADI can reduce the amount of enzyme in the industrial purposes and these results in lowering of production costs. It is observed that the activity of immobilized ADI declines gradually with increasing reuse cycles particularly with chitosan. This decline may be due to the relatively weak binding via non-covalent bonds, the hydrophilic characteristics of chitosan and/or the inhibition of the enzyme by the cumulative products^33^. In addition, the size of bead pores becomes large gradually after repeated use causing enzyme leakage and consequently reduced activity^34^.

The optimal pH for free ADI from *P. chrysogenum* was 6.0, which is similar to the pH of 5.5 reported for the enzyme from *Enterococcus faecalis*^35^. Following immobilization, the ADI’s pH changed from 6.0 to 7.0. This could be because support shields the enzyme from high pH levels. It is possible that changes in the ionization state of the enzyme, which can modify its surface characteristics and cause subunit dissociation and breakdown of the enzyme-substrate intermediate, are the cause of the high acidic or basic pH’s inhibitory influence on enzyme activity. Conversely, the observed improvement in enzyme stability might result from the precise cross-linking achieved during the immobilization of ADI on chitosan, leading to enhanced enzyme stabilization. It has been reported by Atacan et al.^36^ and Khan et al.^37^ that enzyme stability may be increased and conformational stiffness of the three-dimensional structure of the enzyme increased by immobilizing it on the right support using the right technique.

Enzymes are typically sensitive to pH variations. There are several ways in which the pH might affect the activity of enzymes. (1) It may have an impact on the enzyme-substrate complex’s ionization. (2) It may alter how different groups inside the enzyme molecule ionize, which might change how well the enzyme binds to its substrate. (3) It may have an impact on the substrate’s ionization, which may have an impact on how well it binds to the enzyme. (4) It can cause modifications to the protein structure at very high pH values^38^.

The optimal temperature for the free ADI in this study was 40 °C, which is consistent with the temperature reported for the enzyme from *Pseudomonas aeruginosa*^39^.

Because the chitosan blocked the gap between the enzyme molecules to avoid denaturation, the ideal temperature after immobilization was moved from 40 to 45 °C. At the ideal temperature, the kinetic energy will rise in tandem with the pace of the enzyme-catalyzed process. After being incubated at the ideal temperature, the enzyme’s active site will open appropriately for contact with the substrate. Consequently, the activation energy will help the enzyme-substrate complex develop^37^. The chitosan-induced chemical change of the enzyme significantly impacted its heat stability. When it comes to substances that cause conformational changes, such depuration, cross-linking at various structural parts of the enzyme protein will boost structural stiffness and, consequently, protein stability^40^. The increase in reaction rate due to the effect of temperature on the enzyme will eventually be offset by a decrease in reaction rate as the enzyme’s tertiary structure begins to be destroyed^41^. This temperature is sometimes referred to as the optimal temperature, and the activity at that level is defined as the maximum^42^.

The energy required to activate the enzyme (E_a_) was decreased by the immobilization ADI. These findings pertain to the process’s economic viability, and the activation energy (E_a_) is regarded as a crucial factor in estimating the cost of the enzyme^43^. For a chemical reaction to take place, whether it is endothermic or exothermic, the activation energy must be overcome. By binding the reactants in the proper direction to facilitate the reaction, an enzyme can reduce the activation energy of the process^44^.

The Lineweaver–Burk plot, which shows the connection among the concentration of substrate and the rate of an enzyme-catalyzed reaction, was used to calculate the Michaelis constant (K_m_) and maximum velocity (V_max_). The Km values obtained were 0.76 mM for free ADI and 0.87 mM for immobilized ADI. In contrast, ADI of *A. fumigatus* and *A. nidulans* has been found to have greater Km values of 8.8 mM and 4.8 mM, respectively^24,45^. Greater specificity of ADI against L-arginine is shown by the modest K_m_ value.

The immobilized enzyme appears to have a lesser affinity for the substrate than the free enzyme, as shown by the elevated Km value. This outcome is most likely the result of either steric obstruction of the site of activity by the support or diminished enzyme flexibility required for substrate binding^43^. Additionally, the increase in Km value for the immobilized enzyme may suggest that the substrate binds more strongly to the active site compared to the transition state, possibly due to minor conformational changes in ADI^46^.

The following factors make determining the enzyme’s K_m_ value crucial. First, we can determine if the cell requires more enzymes or more substrate for speeding up the process if we can determine the concentration of the substrate in the area of the cell where residues are located^47^. Second, the Km value is an approximate inverse measure of the enzyme’s affinity for catalyzing reactions with two identical substrates; the enzyme prefers to catalyze reactions with the substrate for which it has the lowest K_m_^48^. Thirdly, an approximate assessment of the enzyme-substrate affinity is given by the Km value. For instance, enzymes that catalyse reactions with extremely low concentrations of substrates typically have significantly lower Km values for their substrates, whereas enzymes that catalyse reactions with highly concentrated substrates typically possess relatively high K_m_ values^40^.

In this study, the V_max_ values were 80.0 U mg^− 1^ protein for the free enzyme and 76.3 U mg^− 1^ protein for the immobilized enzyme. The findings show a drop in V_max_ following immobilization, which is typical of enzymatic immobilization and is explained by the enzyme’s structural alterations^49^ and lower accessibility to the substrate^50^. Lower V_max_ values (28.7 and 59.5 U mg^− 1^ protein) of arginine deiminase were reported for the free enzyme from *A. fumigatus* and *A. nidulans*, respectively^24,45^.

The storage stability of free and immobilized ADI was assessed at 4 °C and 25 °C. The results revealed a continuous decline in ADI activity at 4 °C as the storage period progressed. After 30 days of storage, the residual activities were 40% for the free ADI and 84% for the immobilized ADI. Additionally, the residual activity of ADI decreased continuously at 25 °C as the storage period increased. After 30 days of storage, the residual activities were 10% for the free ADI and 75% for the immobilized ADI. Protein-protein interactions in the immobilized form of the ADI may be responsible for its increased stability, since they probably aid in preventing enzyme autolysis. It is crucial to remember that the immobilization procedure does not always guarantee that the enzyme is oriented correctly on the support^51^.

Substrate attachment to the immobilized molecules of enzyme may be hindered by improper fixing and/or modifications to the active sites’ characteristics. Immobilization often increases the rigidity of the enzyme, which is generally associated with enhanced stability^40^. The immobilization of the enzyme on chitosan is probably intended to protect the tertiary structure of the enzyme against conformational changes that result in environmental influences.

However, Naeem et al.^52^ identified two primary effects of trifluoroethanol (TFE) on peptides and proteins: first, TFE stabilizes helical, *β*-turn, and *β*-hairpin structures by promoting intramolecular hydrogen bonding; second, it disrupts the native tertiary structure of intact proteins, leading to increased hydrophobic interactions and the formation of aggregates. Additionally, the impact of TFE on human serum albumin (HSA) provided insights into the conformational changes that occur in HSA during ligand transport across biological membranes, as demonstrated by Naeem et al.^53^.

Compared to the free enzyme, the immobilized ADI demonstrated greater stability against proteases such as trypsin and pepsin. There are two possible causes for the rise in ADI stability proteolysis. The first is the steric impact of immobilization, which delayed the protease’s interaction with the immobilized enzyme and so stopped the proteolysis reaction. Second, it’s likely that immobilization changed the lysine residues of ADI, preventing a certain proteolysis process from taking place on the peptide bonds made up of lysine residues^54^.

The present results show that ADI had minimal effect on the normal cell line (Wi-38) but inhibited the growth of cancer cell lines (Hep-G2 and MCF-7) compared to doxorubicin, the standard drug. These findings suggest that ADI from *P. chrysogenum* could be utilized as an anticancer agent. Supporting this, ADI has demonstrated potential as an antitumor agent against hepatocellular carcinomas^55^. Additionally, L-arginine deprivation by arginine deiminase is used to selectively induce tumor cell death without harming normal cells^56^. Furthermore, ADI induces apoptosis in MCF-7 cells through the activation of the mitochondrial pathway of apoptosis^39^.

## Conclusion and future perspective

ADI was successfully immobilized on chitosan and demonstrated the ability to be reused for 8 cycles with notable stability. The enzyme showed good resistance to stomach proteolytic enzymes. Additionally, the immobilized enzyme-maintained stability for 30 days at both 4 °C and 25 °C, unlike the free enzyme. In future work we must study whether the immobilization process affects the secondary and tertiary structure of the enzyme because this will give clues to know the effect of this process on the enzyme configuration. Additionally, structure and functional relationship must be performed to determine the exact reaction mechanism based on the structure. The enzyme exhibited a significant anti-hepatocellular carcinoma (Hep-G2) with IC_50_ of 69.0 µg mL^–1^ and anti-breast cancer effect with IC_50_ values of 76.8 µg mL^–1^. These results indicate that ADI is a potent anticancer agent. However, further evaluation of its industrial production and in vivo efficacy in animal and human clinical trials is necessary.

## Data Availability

The data that support the findings of this study are available from El-Sayyad but restrictions apply to the availability of these data, which were used under license for the current study, and so are not publicly available. Data are however available from the authors upon reasonable request and with permission of El-Sayyad.
